# A new species of *Stenoloba* Staudinger, 1892 from China (Lepidoptera, Noctuidae, Bryophilinae)

**DOI:** 10.3897/zookeys.310.5125

**Published:** 2013-06-17

**Authors:** Oleg Pekarsky, Aidas Saldaitis

**Affiliations:** 1 H-1068 Budapest, Felsőerdősor u. 16-18, Hungary; 2 Nature Research Centre, Akademijos str. 2, LT–08412 Vilnius-21, Lithuania

**Keywords:** Lepidoptera, Noctuidae, *Stenoloba*, new species, China

## Abstract

A new species of *Stenoloba* from the *olivacea* species group, *Stenoloba solaris*, **sp. n.** (Lepidoptera, Noctuidae), is described from Yunnan, China. Illustrations of the male holotype and its genitalia are provided. A diagnostic comparison is made with *Stenoloba albistriata* Kononenko & Ronkay, 2000, *Stenoloba olivacea* (Wileman, 1914), and *Stenoloba benedeki* Ronkay, 2001 (Fig. 4).

## Introduction

*Stenoloba* Staudinger, 1892 is an East Asian genus of the subfamily Bryophilinae. The first comprehensive revisions of the genus were published by Kononenko and Ronkay (2000, 2001) and Ronkay (2001) based on the East Palaearctic and northern Oriental species.

Subsequently, several articles have increased taxonomic knowledge of this large and very diverse genus including most notably a 2010 publication by Behounek & Kononenko which listed 75 species arranged into 14 species-groups. Recently an additional three new species were described from the Oriental region (Pekarsky 2011), (Sohn and Tzuoo 2012), (Pekarsky et al. in press).

Descriptions of Chinese *Stenoloba* have rapidly increased in the last two decades. Chen (1999) listed only seven species from China, whereas a more comprehensive review of the Chinese *Stenoloba*, published by Han and Kononenko (2009), contained 37 species. This paper describes one more new species and found with the recent and intensive exploration of the Chinese insect fauna and further future discoveries are predicted.

During a spring expedition to north-west Yunnan, a striking specimen of an undescribed *Stenoloba* was collected. The new species, described below, resembles members of the *Stenoloba olivacea* species-group, especially *Stenoloba albistriata* Kononenko & Ronkay, 2000, *Stenoloba olivacea* (Wileman, 1914) and *Stenoloba benedeki* Ronkay, 2001, but displays clearly recognisable external and genital differences.

**Abbreviations of material depositories:** GBG/ZSM = Gottfried Behounek (Grafing, Germany)/Zoologische Staatssammlung, München (Germany); HNHM = Hungarian Natural History Museum, Budapest (Hungary); ZFMK = Zoologische Forschungsistitut und Museum Alexander Koenig (Bonn, Germany).

## Systematic accounts

### Stenoloba solaris sp. n.

urn:lsid:zoobank.org:act:80CD3AD9-74EC-411B-8ECD-BF069AF612A3

http://species-id.net/wiki/Stenoloba_solaris

Figs 1 , 6, 7

#### Type material.

**Figures 1–5.**
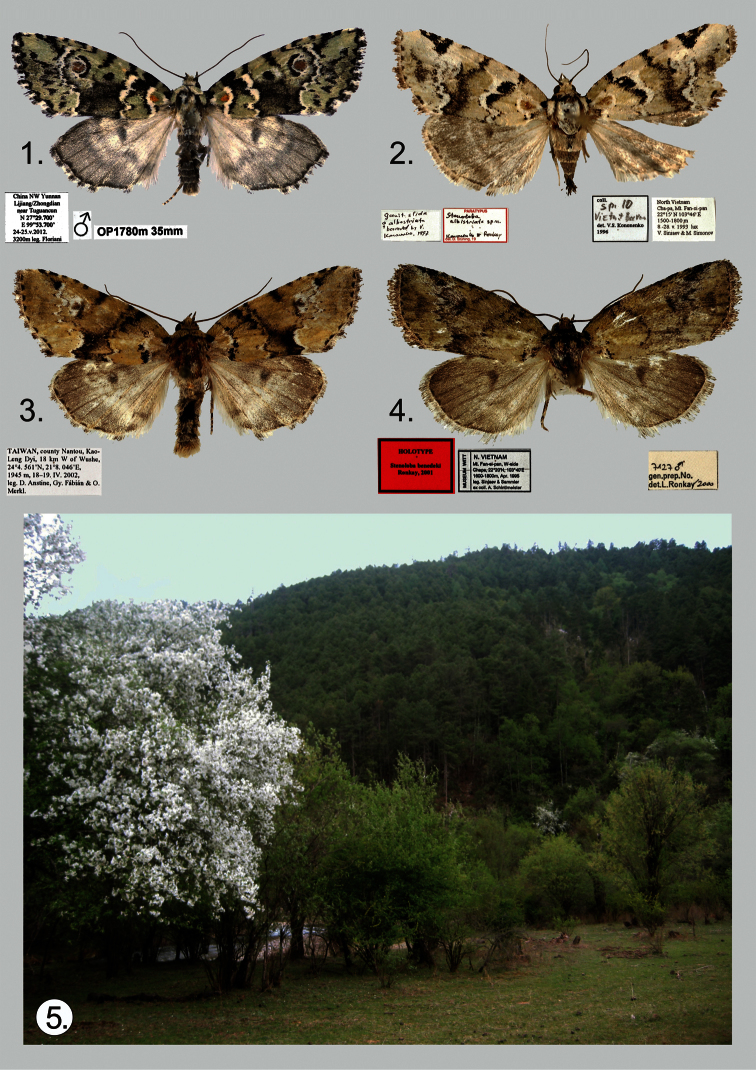
*Stenoloba* spp. adults and biotope. **1** *Stenoloba solaris*, sp. n., male, holotypus, Yunnan, China (GBG/ZSM) **2** *Stenoloba albistriata*, male, paratypus, N. Vietnam (ZFMK) **3** *Stenoloba olivacea*, male, Taiwan (HNHM) **4** *Stenoloba benedeki*, male, paratypus, N. Vietnam (HNHM) **5** Type locality of *Stenoloba solaris*, sp. n. China, NW Yunnan, Lijiang/Zhongdian near Tuguancum, 27°29'700"N, 99°53'700"E.

**Holotype** Male (Fig. 1). China, NW Yunnan, Lijiang/Zhongdian near Tuguancum, 27°29'700"N, 99°53'700"E, 24–25.V.2012, 3200 m, leg. A. Floriani; slide No.: OP1780m (coll. GBG/ZSM).

#### Etymology.

The name “*solaris*” refers to the orange circular patch at the reniform stigma resembling the rising sun.

#### Diagnosis.

**Figures 6–13.**
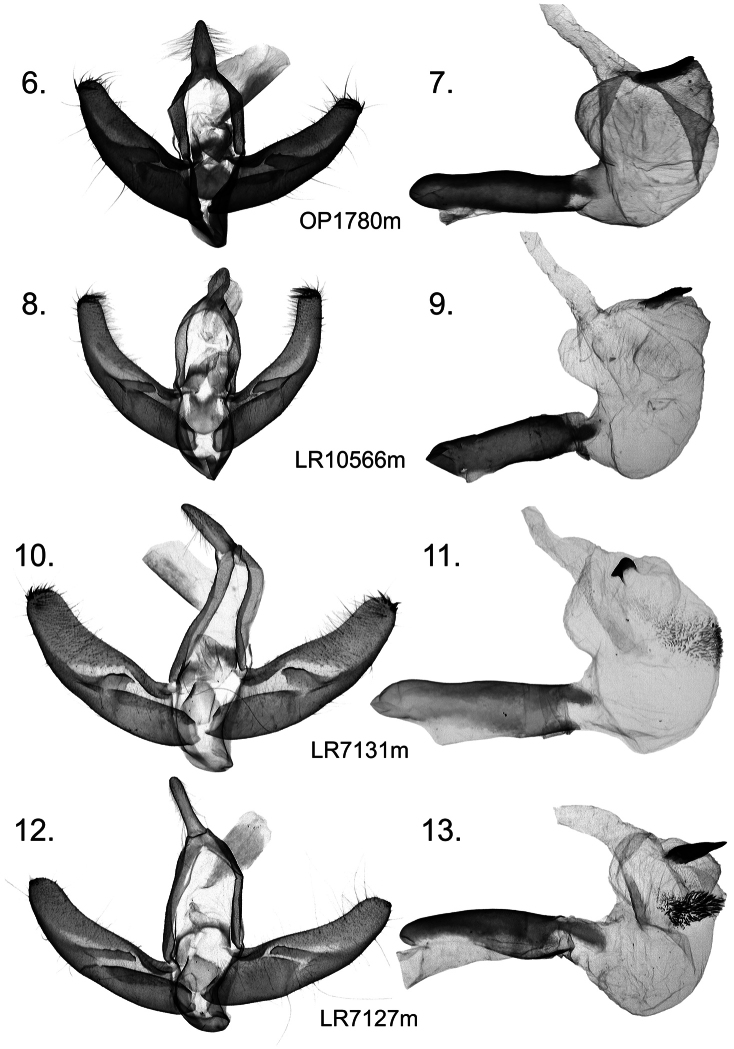
*Stenoloba* spp. male genitalia. **6** *Stenoloba solaris*, sp. n., male, China, capsule, prep. OP1780m **7** *Stenoloba solaris*, sp. n., male, China, aedeagus, prep. OP1780m **8** *Stenoloba albistriata*, male, Vietnam, capsule, prep. LR10566m **9** *Stenoloba albistriata*, male, Vietnam, aedeagus, prep. LR10566m **10** *Stenoloba olivacea*, male, Taiwan, capsule, prep. LR7131m **11** *Stenoloba olivacea*, male, Taiwan, aedeagus, prep. LR7131m **12** *Stenoloba benedeki*, male, Vietnam, capsule, prep. LR7127m **13** *Stenoloba benedeki*, male, Vietnam, aedeagus, prep. LR7127m.

The new species belongs to the *olivacea* species-group and externally resembles *Stenoloba albistriata* (Fig. 2) and *Stenoloba olivacea* (Fig. 3), but is clearly separable from them by both wing pattern and genitalia. The most prominent distinguishing feature, unique within the genus, is the presence of circular orange patches in the forewing basal area and in the reniform stigma. *Stenoloba solaris* differs from all related species by its forewing’s bright lettuce-green colour, as opposed to the olive ground colour and dark grey hindwings of the other species. The specific features of the male genitalia can be found in the shape of the uncus, juxta, and valvae, and in the structure of the vesica. The male genitalia of *Stenoloba solaris* (Figs 6, 7) differ from those of *Stenoloba albistriata* (Figs 8, 9) by the wider base of the uncus, the wider, shorter and less curved valvae, and by the rounded juxta with straight lateral margins. The uncus of *Stenoloba albistriata* is constricted at the base and dilated medially, the longer and narrower valvae have more curved costal margins, and the juxta has concave lateral margins. The other somewhat similar species, *Stenoloba olivacea* (Figs 10, 11) and *Stenoloba benedeki* (Figs 12, 13) each have a longer uncus, rounded juxta, and a large, medially positioned cornuti field consisting of fine spiculi and the terminal cornutus is either small and nail-like (*Stenoloba olivacea*) or large and thorn-like (*Stenoloba benedeki*). In addition, the clasping apparatus of the latter two species is significantly larger than in the new species, but the size of the aedeagus and vesica is practically the same.

#### Description.

Male (Fig. 1). Wingspan 34 mm. Head and thorax lettuce green; collar with a row of black scales at base forming black line; tegulae edged by black line; abdomen blackish grey; forewing relatively elongated, with costa remarkably arched, apex finely pointed, outer margin oblique, ground colour lettuce green with dark-grey area medially; wing pattern well marked with well-developed cross-lines; basal field with circular orange patch bordered with white fascia distally; cross-lines black, basal line strongly marked; subbasal line strong, curved, bordered by white fascia proximally; antemedial line waved, oblique with wide white fascia; lower part of medial area dark grey; medial line nearly straight, slightly bent in middle; postmedial line undulate with white fascia; subterminal and terminal lines formed by large black arrowheads. Noctuid maculation typical and well developed; large orange reniform patch, rounded, defined by black scales; inner edge of stigma forming prominent semilunar arch; orbicular stigma black, dot-like; claviform stigma present as diffuse dark streak; cilia dark grey checkered with white. Hindwing grey, discal spot dark grey, terminal line heavy black. Female unknown. **Male genitalia** (Figs 6, 7). Uncus short and strong, wide at base and tapering towards apex; tegumen somewhat shorter than vinculum; transtilla relatively wide; juxta wide, rounded quadrangular with triangular cleft on posterior margin; vinculum strong, V-shaped; valva simple, elongate, evenly tapering distally and apex rounded, with a few short spine-like setae at apical margin; sacculus elongate, broad; costa slightly concave; clasper forming long, narrow, dorsally dentate plate. Aedeagus short and straight; vesica bulb-like, everted posteriorly, recurved ventrally; medial part of vesica with three diverticula, one with large, stout cornutus.

#### Biology and distribution.

The single male was collected at ultraviolet light on 24–25 May 2012 near Zhongdian in northwest China’s Yunnan province in the remote Baima Xue mountain range (Fig. 5). The new species was collected at an elevation of 3200 meters in a wide river valley near mountain mixed forests dominated by various conifer trees, bushes and rhododendron. Many other spring Noctuidae species were collected there at that time including *Panolis pinicortex* Draudt, 1950, *Orthosia reserva* Ronkay, Ronkay, Gyulai & Hacker, 2010 and *Hada antonraui* Gyulai, Ronkay & Saldaitis, 2011.

## Supplementary Material

XML Treatment for Stenoloba solaris

## References

[B1] Behounek G Kononenko VS (2010) Fourteen new species of the genus *Stenoloba* Staudinger, 1892 from South East Asia (Lepidoptera: Noctuidae, Bryophilinae). Zootaxa 2679: 1-31

[B2] Chen YX (1999) Lepidoptera, Noctuidae. In: Zhu HF et al. (Ed) Fauna Sinica Insect, Vol. 16. Science Press, Beijing, 1596 pp.

[B3] Han HL Kononenko VS (2009) A review of the genus *Stenoloba* Staudinger, 1892 from China, with description of 6 new species and 7 new records for China (Lepidoptera: Noctuidae, Bryophilinae). Zootaxa 2268: 1-22 10.11646/zootaxa.4388.3.1 29690439

[B4] Kononenko VS Ronkay L (2000) A revision of the genus Stenoloba Staudinger, 1892 (Lepidoptera, Noctuidae, Bryophilinae) with description of 25 new species and 3 new subspecies from East Asia (I). Insecta Koreana, 17 (3): 137-174

[B5] Kononenko VS Ronkay L (2001) A revision of the genus Stenoloba Staudinger, 1892 (Lepidoptera, Noctuidae, Bryophilinae) with description of 15 new species and 3 new subspecies from East Asia (II). Insecta Koreana, 18 (2): 95-121

[B6] Pekarsky O (2011) A new Stenoloba Staudinger species from China (Lepidoptera, Noctuidae, Bryophilinae). ZooKeys 108: 67-72.10.3897/zookeys.108.1208 21852929 10.3897/zookeys.108.1208 PMC3119317

[B7] Pekarsky O Dvořák M Ronkay G (in press) Three new species of *Stenoloba* Staudinger, 1892 from Southeast Asia (Lepidoptera, Noctuidae, Bryophilinae). Fibigeriana Supplement Vol. I. Heterocera Press, Budapest.

[B8] Ronkay L (2001) New *Stenoloba* Staudinger, 1892 species from Taiwan and Vietnam (Lepidoptera: Noctuidae, Bryophilinae). Annales Historico-Naturales Musei Nationalis Hungarici, Vol. 93, Budapest 2001, 219–229.

[B9] Sohn JC Tzuoo HR (2012) Two new species of *Stenoloba* (Lepidoptera: Noctuidae) from East Asia with the first description of *S. nora* female genitalia. Journal of Asia-Pacific Entomology 15: 241-244.10.1016/j.aspen.2011.11.005

